# Toward Breeding by Gene Design: Constructing the Ideotype of *Sorghum* (*Sorghum bicolor* (L.) Moench) Adapted for Modern Agricultural Production

**DOI:** 10.3390/plants15101445

**Published:** 2026-05-09

**Authors:** Fei Li, Lingyue Shi, Ji Zhang, Yuli Xiao, Yamei Li, Jianshuang Zhou, Shaoxiong Liu, Shanben Liu, Ruirui Li, Shanshan Wei, Zhi Wang, Guiying Li, Baoqing Dun

**Affiliations:** 1 Zhongyuan Research Center, Chinese Academy of Agricultural Sciences, Xinxiang 453500, China; 18846178408@163.com (F.L.); zhangji@caas.cn (J.Z.); xiaoyl0124@163.com (Y.X.); liruirui24@henu.edu.cn (R.L.); 15633691900@163.com (S.W.); 2 Institute of Crop Sciences, Chinese Academy of Agricultural Sciences, Beijing 100081, China

**Keywords:** sorghum, ideotype, molecular mechanisms, gene design breeding, mechanized production

## Abstract

Sorghum (*Sorghum bicolor* (L.) Moench) is an essential food, forage, and bioenergy crop that plays an irreplaceable role in modern agricultural supply systems and daily life. However, the traditional cultivation varieties, characterized by tall stems, low planting density and large panicles, are incompatible with the requirements of modern intensive agriculture for high-density planting, mechanized harvesting, and efficient resource utilization. Therefore, cultivating an ideotype suitable for mechanized harvesting is the most urgent and practical need for sorghum breeding. This paper systematically reviews the key components of the sorghum ideotype and their physiological basis, focusing on traits such as canopy structure, stalk characteristics, panicle traits, and root systems. Then, the major genes and molecular mechanisms that regulate plant height, stem strength, leaf morphology, and panicle type are described in detail. Additionally, current breeding challenges, including gene pleiotropy, trade-offs among traits, narrow genetic diversity, and limitations in phenotypic identification techniques, are summarized. Finally, we propose modern breeding strategies involving multi-omics approaches, high-throughput phenotyping, gene editing, and computational modeling to advance sorghum breeding into the design era. This will enable the simultaneous improvement in light use efficiency, lodging resistance, and adaptation to mechanized production.

## 1. Introduction

**Figure 1:**
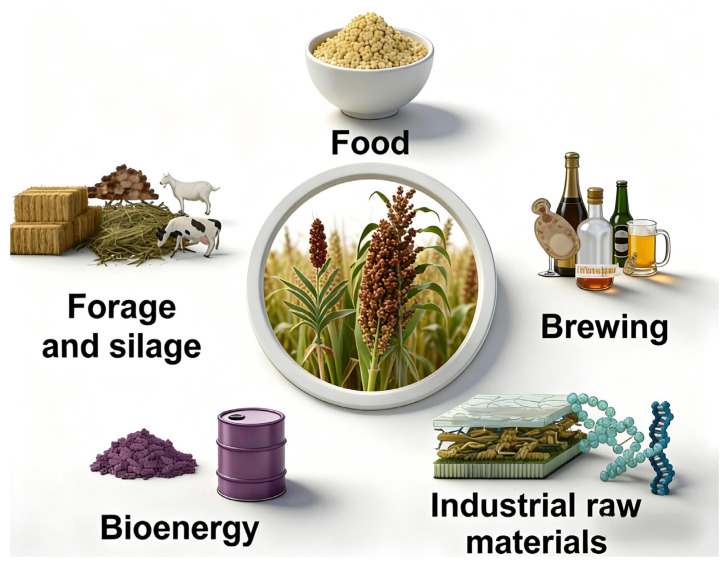
Schematic diagram of the multiple uses of sorghum worldwide (modified from [21]).

Sorghum (*Sorghum bicolor* (L.) Moench), the fifth most important cereal crop worldwide, has become a representative crop in arid and saline–alkaline regions due to its exceptional tolerance to drought, waterlogging, salinity, alkalinity, and high temperatures [1]. Consequently, it plays an irreplaceable role in the global food supply system [2]. The green revolution significantly increased the yields of staple crops and, together with changes in dietary patterns, led to a gradual decline in the food value of sorghum. However, the upgrading and transformation of the liquor and vinegar industries have led to a steady increase in demand for high-quality brewing sorghum [3]. At the same time, silage, broom, and food-grade sorghum continue to advance, driven by the evolving needs of specialized industries. Furthermore, driven by growing consumer demand for natural, healthy, and nutritious food, sorghum has recently regained its place as a valued component of daily diets (Figure 1).

Internationally, the plant height of grain sorghum is typically maintained below 1.5 m to facilitate mechanical harvesting [4]. By contrast, traditional grain sorghum varieties in China generally exceed 1.7 m in height and have a growth pattern characterized by tall stems and large panicles, which is incompatible with the demands of modern agriculture [5]. High-stem varieties are highly susceptible to lodging, leading to reductions in both thousand grain weight and grain weight per panicle. Large and heavy panicles with loosely filled grains result in increased grain loss during mechanical harvesting [6]. In addition, a dense canopy structure restricts light penetration to the middle and lower leaves, consequently reducing light use efficiency and limiting the yield potential of the population [7]. Although domestic researchers have pursued dwarfing breeding strategies in sorghum [8], existing dwarf hybrids have yet to achieve the coordinated optimization of leaf number, leaf morphology, and panicle type following height reduction. Consequently, increases in planting density remain constrained, while the population leaf area index becomes excessively high. This leads to poor ventilation and light penetration in the lower canopy, as well as a decline in net photosynthetic efficiency, which have ultimately constrained improvements in both biomass and grain yield [9]. To address these challenges, sorghum breeding must shift from single-trait improvement to multi-trait collaborative design. This involves developing an ideotype characterized by semi-dwarf stature, strong and resilient stems, compact panicles, and controllable tillering. Such a strategy would simultaneously enhance biomass and grain yield while improving adaptability to mechanized harvesting and dense planting. This would promote the production of more resource-efficient, mechanization-compatible sorghum [10,11].

The ideotype of a crop refers to a morphological paradigm designed to maximize light use efficiency and yield potential through the optimization of plant-type and physiological traits, thereby providing essential guidance for genetic improvement and breeding by design [12,13]. Researchers have conducted long-term yet fragmented investigations into the relationship between sorghum plant type and yield, examining factors such as leaf morphology, panicle structure, stem type, and plant height [14,15,16,17]. Inspired by the green revolution, reducing plant height to enhance lodging resistance and grain yield has become a major trend, making dwarfing breeding in sorghum an effective strategy for mechanized cultivation and simultaneously improving yield and quality [18,19]. However, compared with other staple crops, research on the sorghum ideotype has progressed slowly, and the definitive phenotypic characteristics of the sorghum ideotype remain to be clearly established [20,21].

Developing an ideotype has become an important goal in sorghum breeding. This approach involves integrating high photosynthetic efficiency at the population level, strong lodging resistance, and adaptability to mechanized harvesting through the coordinated optimization of plant morphology. Given this goal, a systematic review of the key traits underlying sorghum ideotypes, their genetic regulatory mechanisms, and current breeding progress is needed. This review should also explore the potential of applying gene design breeding strategies to guide future sorghum improvement.

## 2. Physiological Components of the Sorghum Ideotype

Plant type can be dissected into stem type, leaf type, panicle type, and root type, encompassing key traits such as plant height, stem diameter, tiller number, leaf morphology and spatial distribution, panicle length, grain number per panicle, and root system type. Collectively, these traits determine stem strength, canopy structure, light distribution and interception, and overall resource use efficiency [22,23].

### 2.1. Canopy Structure and Light Capture

Canopy structure plays a critical role in determining the light utilization efficiency of crops. It is fundamentally governed by plant morphology, with factors such as leaf inclination angle, leaf area index (LAI), planting density, and spatial arrangement collectively influencing light interception and distribution within the canopy [24,25].

In sweet sorghum, genotypes with smaller leaf inclination angles enable greater transmission of photosynthetically active radiation (PAR) to the middle and lower canopy layers, thereby increasing the photosynthetic rate of the canopy and its biomass yield [26,27,28]. Leaf area index (LAI) is another critical factor influencing light interception capacity. In sweet sorghum, canopy PAR interception approaches saturation when the LAI increases to 5–6. Beyond an LAI of 6, PAR transmittance drops sharply to below 1%, with most of the light energy being intercepted by the upper canopy, leading to inadequate light availability for lower leaves [29]. Therefore, optimizing light distribution within the canopy by improving the LAI, enhancing photosynthetic efficiency per unit leaf area, and maintaining an appropriate leaf inclination angle distribution can effectively enhance radiation use efficiency and represent a key strategy for improving crop productivity [30].

The ideal plant type for tolerance to dense planting does not require all leaves to be compact; instead, it demands a “smart canopy” characterized by compact upper leaves and relatively relaxed middle and lower leaves. This configuration optimizes light distribution, reduces light saturation in the upper canopy, and enhances photosynthetic activity in the lower layers [31]. In maize, the leaf angle architecture of the smart canopy features erect upper leaves, moderately erect middle leaves, and relatively flat lower leaves, a configuration that boosts yield under high planting densities [32]. However, to date, no gene controlling the smart canopy in sorghum has been identified.

### 2.2. Stem Characteristics and Lodging Resistance

Dwarfing breeding in sorghum is an important strategy for improving lodging resistance, but it requires systematically balancing the relationships among plant height, stem structure, and biomass yield [33,34,35]. Breaking resistance is a comprehensive indicator for evaluating stem lodging resistance, influenced by plant height, internode structure, stem diameter, and lignin content [34]. Plant height exhibits a significant positive correlation with lodging risk. Taller plants have a higher center of gravity and lower breaking resistance, making them more susceptible to lodging. However, excessive dwarfing may lead to reduced biomass yield, underscoring the need for optimization through integration with other stem traits [36]. Internode length and strength are negatively correlated. Excessively long internodes reduce stem rigidity, whereas shortening basal internodes improves breaking resistance [35,37]. Stem diameter contributes to lodging resistance through an increased section modulus. However, thick-stemmed varieties often exhibit uneven lignin distribution, which may compromise structural integrity. Therefore, appropriate cultivation management or targeted varietal selection are necessary to optimize the internal structure of stems and improve material uniformity, thereby enhancing lodging resistance while maintaining overall agronomic performance [38,39].

Lignin is a core component of plant cell walls and, together with cellulose and hemicellulose, constitutes the structural framework of mechanical tissues. Its content and distribution uniformity directly determine stem strength. Sorghum with strong lodging resistance exhibit significantly higher lignin content compared with lodging susceptible materials [35,40]. Genes involved in lignin biosynthesis, such as *SbCAD* and *SbCCoAOMT*, co-localize with lodging resistance QTLs, suggesting that enhancing these pathways can improve stem rigidity [36]. Breaking resistance is closely correlated with stem stiffness, and predictive models that incorporate internode density and volume enable the accurate screening of lodging-resistant genotypes [39]. The optimal lignin level depends on the target end-use in sorghum breeding: higher lignin is preferred for grain types that require strong stalks, whereas lower lignin is suitable for forage types where digestibility is prioritized [41]. In forage sorghum, the brown midrib (BMR) trait conferred by mutations in lignin biosynthesis genes such as *bmr6* and *bmr12* reduces lignin content by 20–40% and increases neutral detergent fiber digestibility from 47.37 to 54.98% [42] However, brown midrib lines often exhibit reduced biomass yield and increased lodging susceptibility. Importantly, recent studies show that negative traits, such as height reduction, delayed flowering, and lodging, are not universally observed [43]. For brewing sorghum, lignin content influences processing efficiency by affecting starch accessibility and steam explosion efficiency. Lower lignin improves saccharification yields but may compromise stalk strength [44].

**Figure 2:**
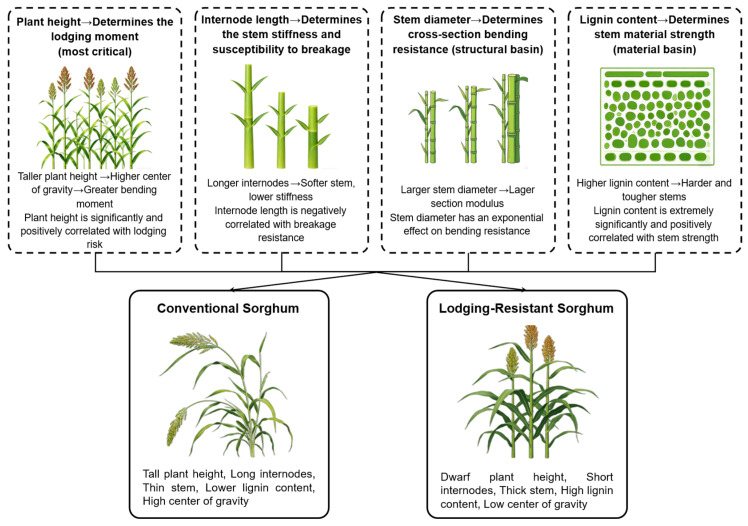
Physical relationship model of plant height, internode length, stem diameter, lignin content, and stem lodging resistance in sorghum.

Dwarfing in sorghum should avoid excessive height reduction; instead, lodging resistance can be enhanced by shortening the basal internodes, increasing the stem diameter, and promoting lignin deposition (Figure 2). These modifications should be coordinated with improvements in canopy structure and resource allocation to achieve a population type characterized by dwarf yet robust stems and dense yet well-ventilated canopies, thereby maintaining biomass yield while ensuring lodging resistance [36,37]. Achieving an optimal balance between dwarfing and biomass yield requires multi-dimensional regulation. Dwarfing genes such as *Dw1* to *Dw4* can be utilized to reduce plant height [45], while canopy structure can be optimized using *StgQTLs* to minimize unproductive tillers and enhance photosynthate accumulation [46]. In addition, optimizing planting density helps to coordinate light penetration with stem robustness, and appropriate nitrogen application facilitates effective lignin deposition [31,47]. Integrating lodging-related QTLs identified through genome-wide association studies (GWASs) with lignin biosynthesis genes and root-type regulators enables the development of dwarf, strong-stem, deep-root ideotypes [33,40,47].

### 2.3. Panicle Traits and Sink Capacity

The coordination of panicle traits and canopy structure is essential to efficient grain filling, and achieving source–sink balance requires optimization across multiple traits [48]. The compatibility between panicle type and canopy type serves as the foundation for this balance. Compact panicle types, which are characterized by dense primary branches and numerous rachis nodes, enhance light utilization at the population level, improve canopy ventilation and light penetration, and ensure alignment between panicle sink strength and canopy source capacity [49,50,51].

Superior hybrids with desirable performance typically exhibit moderate panicle types and compactness, in which panicle length and width are well matched with the canopy leaf area index to promote dry matter partitioning during grain filling [51,52,53]. Peduncle length plays a key role in influencing the spatial positioning of the panicle. An optimal peduncle length positions the panicle within the well-illuminated upper-to-middle canopy, thereby reducing leaf shading [53,54]. There is a trade-off between grain number per panicle and thousand grain weight, which is largely mediated by canopy photosynthetic capacity. Research found that although triple grain mutants exhibited a significant increase in grain number per panicle, thousand grain weight declined by 41% [55], and the proportion of non-grain panicle biomass increased, indicating that the supply of canopy photosynthates was insufficient to meet the filling demands of the excessive grains. However, a well-designed canopy structure can help mitigate this trade-off. Notably, stay-green QTLs (*Stg1*–*Stg4*) enhance post-anthesis source capacity by extending photosynthetic duration, optimizing canopy architecture, and improving water use efficiency during grain filling [56]. Near-isogenic-line studies have demonstrated that *Stg* QTLs increase post-anthesis biomass production and grain yield under both drought and well-watered conditions, with no consistent yield penalty [46]. These traits could theoretically mitigate the sink–source imbalance in high-grain-number mutants by providing additional assimilates to support grain filling [57]. It was found that the leaf primordia elongation rate is coordinated with the timing of panicle differentiation [58]. Specially, panicle differentiation is initiated once the canopy leaf area reaches a threshold, ensuring that photosynthate supply during grain filling matches grain demand. From a genetic improvement perspective, selecting parental lines with stable inheritance of primary and secondary branch numbers enables the development of combinations in which grain number per panicle and thousand grain weight are synergistically enhanced [53]. In addition, optimizing planting density helps to establish a favorable canopy structure that maximizes grain-filling efficiency [59].

### 2.4. Root System Architecture and Flow Support

The capacity of root systems to anchor and efficiently take up water and nutrients is fundamental to stress adaptation and yield maintenance of plants, and these functions are closely regulated in relation to aboveground traits associated with lodging resistance and stress tolerance [60]. Root anchoring capacity is largely determined by brace roots and root system type, and the number of brace root nodes is positively correlated with plant height and stem diameter, thereby contributing to enhanced lodging resistance [61]. It was reported that root lodging led to reductions of 40% in biomass and approximately 50% loss in grain yield in mechanically harvested sweet sorghum, highlighting the critical impact of inadequate root anchorage on yield performance [38]. Moreover, nodal roots improve anchorage through deep soil penetration, and these traits are genetically independent of lodging-related stem characteristics, enabling synergistic improvement [62]. The root length density (RLD) estimation model demonstrated that fine roots in sorghum exhibit an isotropic distribution, whereas coarse roots shift from a horizontal to a vertical orientation with the increase in soil depth, a configuration that optimizes nutrient acquisition [63]. And it was further demonstrated that genotypes with steep root angles achieved significantly greater root length density and biomass in the 15–45 cm soil layer than those with shallow root angles [60].

Root function is closely associated with aboveground stress tolerance. Mace et al. (2012) demonstrated that QTLs for nodal root angle co-localize with QTLs for drought tolerance, contributing to drought adaptation by modulating root distribution patterns [1]. Rajkumar et al. (2013) identified QTLs for root volume, fresh weight, and dry weight, which co-localize on chromosome *SBI-04* [64]. These traits were positively correlated with drought tolerance and did not overlap with yield-related QTLs, thereby enabling simultaneous improvement in root traits and stress tolerance without compromising yield potential. Furthermore, there is a balance in resource allocation between roots and aboveground organs. Singh et al. (2011) observed that nodal root angle and plant-type traits are independently inherited, allowing for the improvement in stress tolerance without negatively affecting biomass accumulation [62]. Through brace root anchorage, optimization of deep soil root architecture, and enhanced fine-root function, sorghum roots achieve dual functions of anchoring and nutrient uptake. The synteny of their underlying genetic loci with QTLs for aboveground lodging resistance and drought tolerance provides a promising pathway for synergistic improvement in molecular breeding [65].

### 2.5. Characteristics of the Expected Sorghum Ideotype

The characteristics of ideotypes of staple crops adapted to modern high-density planting and resource-limited conditions have been well characterized [66]. In rice, the ideotype is generally characterized by a moderate plant height of 90–110 cm; 8–10 effective tillers; robust stems; erect, upright and dark green leaves; a compact canopy type; and numerous spikelets per panicle with rapid grain-filling capacity [67]. In wheat, the ideotype is typically defined by a plant height of 70–80 cm; strong stems with short and thick internodes; flag leaves that are short, broad, thick, and dark green; few unproductive tillers; a compact plant type; and large spikes with many grains [68]. The maize ideotype emphasizes a smart canopy structure, characterized by upright leaves above the ear, longer and more horizontally oriented leaves in the middle canopy, and horizontal leaves in the lower canopy to optimize light utilization, which is complemented by a reduced plant height of 2.2–2.5 m, moderate ear placement, thick stalks, and compact tassels [69,70].

Although the definitive phenotypic characteristics of the sorghum ideotype remain to be fully elucidated, several key architectural and physiological traits have reached a broad consensus in recent studies (Table 1). The sorghum ideotype tailored for modern high-density planting and mechanized harvesting represents a synergistically optimized architectural and physiological system [71]. Although improved equipment can be used to mechanically harvest higher sorghum, it is generally believed that the optimal plant height for mechanized harvesting is 120–150 cm (Mace et al., 2012; Hilley et al., 2016 [1,15]), balancing biomass accumulation with harvestability. Varieties exceeding 1.8 m cause header clogging and increased grain loss [72]. The ideotype is characterized by semi-dwarf-to-dwarf stature (1.2–1.5 m), which lowers the center of gravity and improves lodging resistance. It exhibits short, thick basal internodes; large stem diameter; thick rind; and high lignin deposition. This provides a breaking strength > 8.5 N/mm^2^ to withstand mechanical harvest [73]. The canopy is compact, with erect upper leaves and progressively more horizontal lower leaves, coupled with an optimal leaf area index (LAI = 5–6) to maximize light penetration, vertical light distribution, and population-level radiation use efficiency. The panicle is compact to semi-compact, with adequate peduncle exsertion (>15 cm); moderate size; and rapid grain dehydration to minimize shattering loss and enable efficient threshing [74]. The root system features steep angles, abundant brace roots, and deep soil distribution for strong anchorage and reduced belowground competition, supported by stay-green QTLs (*Stg1*–*4*) to sustain post-flowering source supply [60,62]. Collectively, these coordinated traits integrate high light use efficiency, strong lodging resistance, and excellent mechanization adaptability, forming the core architectural foundation for sustainable, high-yield sorghum production.

In addition to the basic architectural traits discussed above, several physiological and agronomic characters serve as valuable selection criteria for high-yielding sorghum ideotypes. Stability in flag leaf size is associated with high and stable grain yields in dual-purpose sorghum [75]. High flag leaf area and leaf dry matter and relative water content can be exploited as morpho-physiological markers for higher grain yield [76]. Leaf greenness (SPAD chlorophyll content) has a positive direct effect on single-plant yield and is a reliable indicator of stay-green expression. The SPAD value shows a significant linear relationship with total leaf chlorophyll and with visual stay-green rating, making it a practical selection criterion for sorghum breeders [77]. Green leaf percentage and the number of green leaves are associated with stay-green and post-flowering drought tolerance, and the number of green leaves has been identified as a favorable trait in dual-purpose sorghum genotypes combining grain and biomass production [76]. Positive and significant correlations of grain yield with leaf area index and stay-green have been observed under drought stress. Finally, genotype-by-trait analysis revealed strong correlations between grain yield and panicle width, indicating the possibility of indirectly selecting for grain yield by using panicle width [78]. Collectively, these traits offer practical, complementary criteria for screening high-yielding sorghum lines across diverse environments.

The sorghum ideotype differs significantly from those of rice, wheat, and maize, necessitating a dedicated framework rather than direct extrapolation from other cereals. On the one hand, multiple end-use types (grain, sweet, forage, and broom) demand tailored ideotype configurations. On the other hand, a wide optimal plant height range (1.2–1.5 m) balances lodging resistance, biomass accumulation, and mechanized harvestability, making sorghum taller than rice and wheat but shorter than traditional maize. Furthermore, the sorghum ideotype described in this review is primarily tailored to high-input, mechanized production systems in optimal environments. However, as many sorghum-growing regions are semi-arid or marginal, the ideotype can be adapted by incorporating stress tolerance traits while preserving its core plant architecture. Recent studies on recommended wheat varieties have shown that they do not fully match the ideotype characteristics, suggesting that the concept may not be universal or that other conflicting factors may prevent ideotype expression [79]. Whilst there are benefits to the concept of the ideotype, the power of the concept is lost when integrating the multitude of factors that, in combination, determine plant structure and function in a given environment. Nevertheless, regardless of these limitations, we argue that there is still a role for the ideotype in crop breeding but that this concept needs to be expanded to emphasize the genetic and environmental interactions that influence plant physiology.

## 3. Key Genes and Molecular Mechanisms Regulating Plant Type

After clarifying the physiological components of the sorghum ideotype, we now examine the genetic and molecular regulators that control these traits. The formation of plant type is fundamentally governed by genetic regulation, with genes serving as the genetic foundation of phenotypic traits. A deep understanding of the key genes and their molecular regulatory mechanisms underlying the sorghum plant type is a critical enabler for breakthroughs in breeding technologies and the acceleration of cultivar development.

### 3.1. Regulators of Plant Height and Stem Strength

Sorghum exhibits a wide range of plant heights, ranging from 0.5 to 6.5 m. The hybrids currently cultivated for production remain generally tall, making them highly susceptible to severe lodging under adverse environmental conditions. Appropriate dwarfing enhances lodging resistance and reduces yield losses while also facilitating dense planting, an essential prerequisite for developing ideotype suitable for mechanized operations. This represents an inevitable trend in promoting the advancement of sorghum toward large-scale and mechanized production [80,81,82]. Approximately 100, 30, and 60 dwarfing-related regulatory genes have been characterized in major staple crops such as rice, wheat, and maize, respectively [83,84,85]. Although considerable research has recently been devoted to understanding plant height in sorghum, the progress in the cloning of dwarfing genes remains limited, significantly lagging behind that of other staple crops.

**Figure 3:**
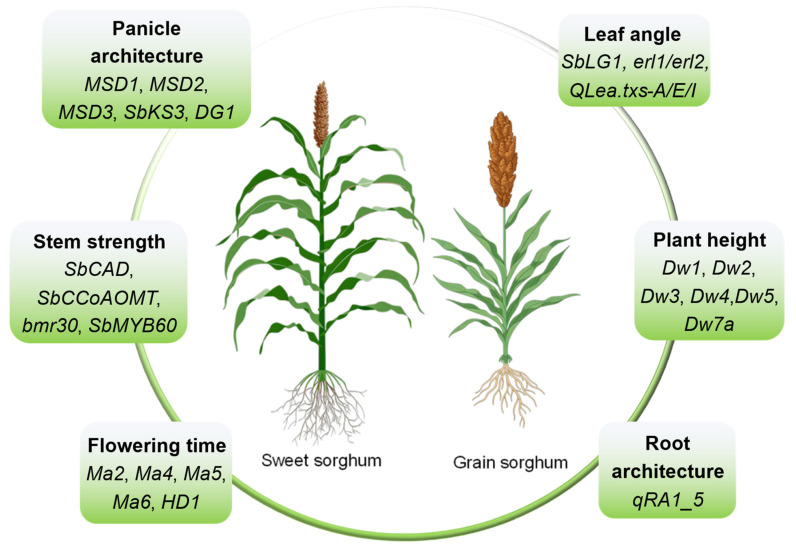
The main favorable alleles associated with plant architecture for molecular design breeding in sorghum.

Point mutations in the *Dw* series of genes constitute an important genetic foundation for dwarfing breeding design in sorghum [86]. Sorghum dwarf is primarily governed by six loci, *Dw1* to *Dw5* and *Dw7a* (Figure 3). Among these, the recessive alleles *dw1* and *dw3* have been widely incorporated into commercial grain sorghum varieties to enable large-scale cultivation [87]. *Dw1* (*Sobic.009G229800*) plays a novel role in brassinosteroid (BR) signaling by encoding a protein involved in the regulation of cell proliferation. This protein interacts with *BIN2* (*BRASSINOSTEROID INSENSITIVE 2*), a negative regulator of the BR signaling pathway, and positively regulates BR signal transduction by inhibiting the nuclear localization of *BIN2* [45]. *Dw2* (*Sobic.006G067700*) encodes a protein kinase homologous to KIPK [88]. *Dw3*/*SbPGP1* (*Sobic.007G163800*) encodes an ABCB1 auxin transporter. Its functional alteration leads to reduced auxin transport in seedlings, resulting in internode shortening, stem thickening, and modifications to the vascular system, ultimately contributing to dwarfing [89]. *Dw4* has been mapped to a 6.6 Mb region on chromosome 6, but the causal gene remains unidentified [4]. A fifth dwarfing locus, *Dw5*, has also been reported [19], along with two marker genes associated with plant height variation (*Sobic.003G119600* and *Sobic.010G085400*) [90]. More recently, *Dw7a*, which encodes an R2R3-type MYB transcription factor, has been identified as a novel plant height locus [91].

Plant height reduction is primarily controlled by the combined effect of major-effect loci (reduce stem height) and minor-effect loci (shorten peduncle length). Among the characterized dwarfing genes, three dwarfing genes, *Dw1*, *Dw2*, and *Dw3*, act synergistically to modulate plant height through modification of internode length [88,92,93]. Among these, *Dw1* and *Dw3* are generally recognized as major-effect loci [94,95]. *Dw4* is thought to influence plant height by modulating peduncle length [4].

The degree of stem fiberization, lignification, and number of vascular bundles influence the integrity and stability of the plant and are closely associated with lodging resistance [96]. These traits are governed by a complex molecular regulatory net involving the coordinated action of multiple genes [97]. Loss-of-function mutations in the *bmr30* gene, which encodes chalcone isomerase (CHI), lead to a significant reduction in lignin content, thereby decreasing stem strength [98]. In contrast, *SbMYB60* has been identified as a positive regulator. Overexpression of *SbMYB60* activates lignin biosynthesis in drought-tolerant sorghum and enhances both stem mechanical strength and stress tolerance [99]. Therefore, appropriately managing stem diameter while concurrently improving stem toughness and supporting capacity can not only enhance lodging resistance but also delay premature senescence, ultimately contributing to improved yield.

### 3.2. The Designers of Leaf Type

Leaf morphology is a key agronomic trait in sorghum, shaped by environmental and genetic regulation factors, playing a fundamental role in determining photosynthetic capacity, plant type, and growth [100]. Among these traits, leaf angle is a primary determinant of canopy compactness, as it governs the spatial arrangement of leaves and directly influences light utilization efficiency at the population level [87]. The molecular regulation of this process has been dissected through QTL mapping, positional cloning, and mutant analysis.

Over 80 QTLs for leaf angle have been identified in sorghum, with several fine-mapped to genes regulating ligular development (Figure 3) [101]. A major QTL on chromosome 7 co-localizes with *Dw3*, which encodes an auxin transporter; reduced auxin transport leads to smaller leaf angles and a more erect canopy [89,102]. Another QTL on chromosome 6 led to the identification of *Sobic.006G247700*, the sorghum ortholog of maize liguleless1 and rice *OsLG1*, which is required for ligule formation; its loss causes erect leaves due to the absence of a bending zone [103,104]. The ligular joint is a specialized structure where the leaf blade bends. Adaxial cell elongation drives leaf inclination, while the abaxial side remains static. Auxin establishes a gradient across the joint, promoting asymmetric expansion. Functional *Dw3* facilitates auxin efflux, causing adaxial accumulation and outward bending (larger angle); loss-of-function reduces auxin, resulting in upright leaves. *Sobic.006G250500*, an ortholog of rice *OsIBH1*, encodes an HLH transcription factor that represses cell elongation in the joint; its overexpression reduces leaf angle [105].

Several sorghum mutants with altered leaf angles have been characterized. *erl1* and *erl2* exhibit upright leaves due to defects in ligular elongation, though the causal genes remain unknown [106]. *SbLG1* mutants completely lack a ligule, producing erect, compact leaves suitable for dense planting [107]. Collectively, leaf angle is determined by auxin-driven asymmetric cell expansion in the ligular joint, modulated by *Dw3*, *SbLG1*, and *HLH* factors, a coherent model that provides clear targets for breeding ideotypes with optimized canopy architecture. Advances in understanding the genetic basis of sorghum leaf angle provide opportunities to explore the potential for improving light interception capacity and canopy photosynthetic efficiency. However, current research into the underlying genetic mechanisms in sorghum remains limited, highlighting the need for further investigation [108].

### 3.3. Determinants of Panicle Type

Panicle-type traits are closely associated with grain yield in sorghum, with the inflorescence structure influencing grain number per panicle being of particular importance for breeding purposes [109]. A suite of related genes has been identified through GWASs (Figure 3). There are *Sobic.001G106200*, *Sobic.002G247800* and *Sb03g030635* for panicle length; *Sobic.003G027000* and *Sobic.010G267700* for panicle width; and *Sobic.001G214000*, *Sb02g042400*, and *Sobic.006G254900* for panicle compactness [52,110]. The *MSD1* (*SORBI_3007G135700*) and *MSD2* (*SORBI_3006G095600*) genes encode a transcription factor containing a TCP domain and a 13-lipoxygenase (LOX) enzyme, respectively, which modulate spikelet fertility in sorghum via the jasmonate pathway, thereby influencing panicle type and yield [111,112]. Another gene, *MSD3* (*Sobic.3001G407600*), encodes a plastidial ω-3 fatty acid desaturase which converts linoleic acid (18:2) into linolenic acid (18:3). This activity regulates the fertility of pedicellate spikelets, ultimately affecting panicle type and grain yield [113].

Additionally, *SbKS3* (*Sb06g028210*) has been identified as a candidate gene regulating seed number in sorghum inflorescences through the gibberellin (GA) biosynthesis pathway [114]. The *DG1* gene, which is associated with double-grain spikelets, significantly influences grain number per panicle and grain yield [115]. *DG1* encodes a protein containing a homeobox domain. Plants carrying *DG1* have slightly smaller grains and lower individual grain weight but have more grains per panicle and higher total grain weight per panicle, with no significant effect on plant height, tillering, nor flowering time. Meanwhile, the plant height control genes *Dw1* and *Dw3* also exert pleiotropic effects on both panicle length and grain weight per panicle [72,89].

### 3.4. Integrators: Hormonal and Photoperiod Pathways

#### 3.4.1. Plant Hormones Coordinate Plant-Type Development

Plant growth and development are coordinately regulated by interactions between hormone contents and signaling pathways [116]. The major plant hormones include gibberellins (GAs), brassinosteroids (BRs), and auxin (Indole-3-acetic acid, IAA). The biosynthesis and signal transduction of GAs and BRs are particularly important for plant-type regulation [117,118].

GAs act as key regulatory hormones in sorghum growth. GAs orchestrate traits such as plant height, tillering, and flowering time by perceiving environmental signals, including photoperiod, planting density, and salt stress, through molecular signaling pathways and hormone interaction networks, thereby enabling sorghum to adapt to complex environments [119,120]. Photoperiod is a critical environmental cue for establishing plant type, with gibberellin levels responding to photoperiod through rhythmic fluctuations in hormone content [121]. This process is closely associated with phytochrome B (phyB). Loss-of-function phyB mutants (ma_3__r_) exhibit early flowering, rapid elongation, and reduced tillering due to aberrant gibberellin signaling while also showing insensitivity to photoperiod variation. These observations provide evidence that phyB is involved in gibberellin-mediated photoperiod signal transduction [122].

In response to high planting density, the expression of *SbGA3ox2* (a GA_3_-oxidase gene) is significantly upregulated in sorghum leaves, leading to increased accumulation of bioactive gibberellins. This upregulation directly induces internode elongation and is accompanied by typical shade avoidance responses, such as increased plant height and enlarged leaf angles, thereby enhancing light capture capacity and improving adaptation to competitive light environments within high-density populations [123]. Gibberellins play a key role in maintaining plant type under salt stress by mitigating stress-induced damage [124]. Sorghum exhibits reduced germination rates and suppressed metabolism under high-salinity conditions. The exogenous application of gibberellins effectively improves seedling leaf morphology, regulates the expression of genes responsive to salt stress, and alleviates the inhibitory effects of salinity on plant type [125]. This regulatory effect relies on crosstalk between gibberellin signaling pathways and stress tolerance metabolic networks, which sustain normal growth and development by activating antioxidant systems and promoting the accumulation of osmotic regulatory substances [124,126].

GAs regulates the sorghum plant type through a derepression mechanism. In the absence of GAs, the protein DELLA binds to and inhibits growth-related transcription factors. However, when GAs bind to the GID1 receptor, they induce the ubiquitination and subsequent degradation of these proteins, thereby relieving growth inhibition [125,127]. This mechanism is particularly critical to regulating plant height. GA content in sorghum stem nodes shows a significant positive correlation with plant height. GA promotes internode elongation through enhanced cell elongation, which is evidenced by the GA level in the stem nodes of tall sorghum genotypes exceeding those in dwarf genotypes [120]. Gibberellin regulation of tillering exhibits concentration-dependent effects. Low concentrations of GAs can inhibit tillering independently, whereas high concentrations promote stem elongation while simultaneously inhibiting tillering [122,128].

Due to its strong pleiotropic effects on plant height, tillering, and flowering time, the GA pathway must be manipulated with caution in ideotype breeding. Complete loss-of-function mutations in GA biosynthesis genes often lead to excessive dwarfing, reduced grain yield, and altered flowering [129]. However, partial loss-of-function alleles (e.g., weak alleles of *SbGA3ox2*) or tissue-specific promoters that restrict GA manipulation to stem internodes could achieve moderate height reduction without compromising tillering or grain filling [122]. Furthermore, the GA pathway’s integration with environmental signals (e.g., photoperiod and density) makes it an attractive target for optimizing plant architecture under changing climates [122]. Future ideotype design should therefore focus on fine-tuning GA signaling rather than complete knockout.

BRs are steroid hormones that systematically coordinate essential traits in sorghum, including plant height, photosynthetic structure, and grain development. They achieve this by precisely perceiving environmental signals, such as salt stress and drought, through core gene regulation and hormone interaction networks. BRs serve as a critical physiological regulatory hub for sorghum adaptation to marginal environments [130,131]. BRs alleviate salt stress damage by remodeling the anatomical structure of leaves. The mechanism involves BRs increasing bulliform cell area to maintain leaf turgor pressure while simultaneously promoting cuticle thickening to reduce non-stomatal water loss and enhance water use efficiency [132]. Furthermore, BRs improve both photosynthetic capacity and water use efficiency by increasing stomatal density and optimizing mesophyll intercellular spaces to reduce CO_2_ diffusion resistance.

Under drought stress conditions, BRs activate stress tolerance metabolic pathways through the regulation of receptor genes [133]. The BR receptor, *SbBRI1*, can be targeted for directed mutagenesis to significantly enhance osmotic stress and drought tolerance in sorghum [134]. The molecular basis of BR-mediated drought adaptation involves the downstream transcription factor *SbBES1*, which binds to promoter elements of genes in phenylpropanoid metabolism. *SbBES1* then enhances drought resistance while maintaining the compact plant type, effectively preventing the excessive elongation or growth inhibition that often accompanies drought stress [135]. This integrated regulatory network thus achieves the synergistic optimization of drought tolerance and plant architecture stability.

The BR pathway offers a more promising target for precise ideotype engineering. The *dw1* dwarfing gene in sorghum activates BR signaling by interacting with and inhibiting the nuclear localization of *BIN2*, a negative regulator of the pathway. This molecular mechanism leads to shortened internode length and reduced plant height with notable tissue specificity, primarily shortening stem height below the peduncle without affecting the normal growth of sweet sorghum hybrids. This highlights the precise adaptability of BRs to diverse sorghum plant types [136]. In addition, BRs regulate grain development through the BZR transcription factor family, further optimizing yield-related plant type [137]. Moreover, BR signaling components such as *SbBRI1* (the BR receptor) and *SbBES1* (a downstream transcription factor) directly link stress tolerance (e.g., drought and salinity) to plant architecture [134]. Directed mutagenesis in *SbBRI1* or *SbBES1* can enhance osmotic stress tolerance while maintaining a compact plant type, as recently demonstrated in sorghum [135].

#### 3.4.2. Genes Involved in the Photoperiod Pathway

The photoperiod pathway serves a critical regulatory route controlling flowering time in sorghum, and understanding how photoperiod-related genes modulate flowering is essential to deciphering the regulation of sorghum plant type [138,139]. Studies have revealed that the *CONSTANS* (*CO*) and *FLOWERING LOCUS T* (*FT*) genes are the most important regulators in this pathway. *CO* encodes a zinc finger transcription factor, while *FT* encodes an RAF kinase-related protein. Together, these genes integrate light signals and circadian timing to control flowering initiation in response to photoperiod [140,141].

Sorghum is a short-day C4 plant characterized by pronounced photoperiod sensitivity. Photoperiod genes regulate flowering time, thereby indirectly influencing plant height and biomass allocation [50,142]. Several photoperiod responsive genes have now been cloned in sorghum, among which the *Ma1*–*Ma6* gene family constitutes the key photoperiod regulatory network, primarily controlling the transition from vegetative to reproductive growth. This gene family plays a critical role in plant type [143,144]. As a key flowering repressor, *Ma1/SbPRR37* delays flowering by activating *SbCO*, suppressing *SbEhd1*, and downregulating *FT* genes such as *SbCN8* and *SbCN12* [141,145,146]. But its effect on flowering time differs dramatically between long-day (LD) and short-day (SD) conditions due to a light-dependent, clock-regulated expression pattern. Thus, the same genetic module (Ma1/SbPRR37) delays flowering in LD but permits rapid flowering in SD, directly shaping the contrasting plant architectures required for biomass vs. grain production [146]. *Ma2* encodes an *SYMD* domain containing methyltransferase and enhances the expression of *Ma1* and *SbCO*, synergistically repressing flowering [147]. In contrast, while the precise function of *Ma4* remains unclear, it is known to interact with *Ma2* and influences photoperiod perception by modulating the expression of *SbPRR37* and *SbCO* [141]. The phytochrome genes *Ma3/PHYB* and *Ma5/PHYC* encode *PhyB* and *PhyC*, respectively. *PhyB* represses flowering by regulating genes including *SbPRR37* and *SbGHD7*, while *PhyC* is essential to far-red light sensing and flowering under long-day conditions [148]. *Ma6/SbGhd7* acts as a negative regulator of flowering, which delays flowering under long-day conditions [144,149].

In addition, the *CONSTANS* family gene *HD1* delays flowering under long-day conditions due to a 5 bp deletion [150]. Other genes, such as *SbSUC9* and *SbMED12*, have also been implicated in photoperiod regulation [151], while genes including *ELF* and *ESD* can suppress photoperiod sensitivity. Together, these genes form a multi-level regulatory network that precisely interprets photoperiod signals, ultimately shaping the sorghum plant type.

## 4. Practices and Challenges of Sorghum Breeding

Understanding the genetic basis of plant architecture provides the foundation for assessing current breeding efforts and their limitations. Notable progress has been achieved in sorghum breeding in China [3,9,10,11]. However, further breakthroughs in breeding are constrained by several factors, such as the pleiotropic effects of genes, the negative correlations among agronomic traits, the relatively narrow genetic base of germplasm resources, and the lack of advanced phenotyping technologies [21].

### 4.1. Evolution of Sorghum Plant-Type Breeding

**Figure 4:**
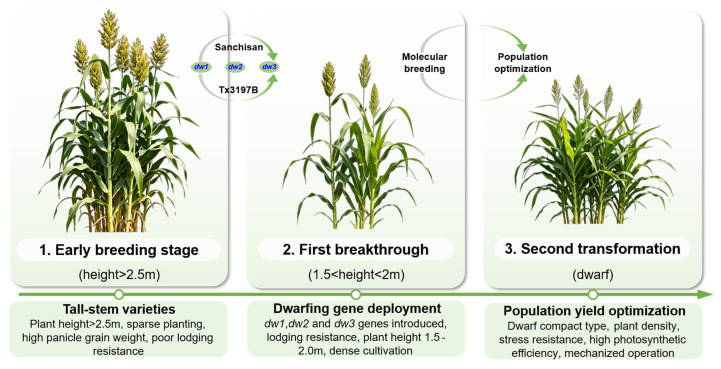
Schematic illustration of the transition from tall to dwarf plant types and the associated gene introgression in sorghum breeding for plant architecture.

The sorghum breeding has undergone a strategic transformation from traditional tall-stem varieties to modern medium and dwarf stem types and from optimizing the yield of individual plants to optimizing population-level yield (Figure 4). Early sorghum breeding in China was characterized by tall-stem varieties, with plant heights of mainstream cultivars typically exceeding 2.5 m. These varieties accumulated substantial biomass through well-developed individual vegetative organs, which adapted to traditional sparse planting patterns [8,9,10]. However, despite high grain weight per panicle, they exhibited several drawbacks, including poor lodging resistance, inadequate ventilation and light penetration, and difficulties associated with manual harvesting. Moreover, population density was limited, hindering the realization of yield potential at the population level [152,153].

The first breakthrough in sorghum plant-type breeding was achieved through the discovery and deployment of dwarfing genes (Figure 4), which drove the transition from tall to medium and dwarf varieties [3]. Through hybrid breeding, researchers integrated elite dwarfing sources from both domestic and international germplasm, combining dwarfing genes from the Chinese restorer line Sanchisan with those from the foreign sterile line Tx3197B to clone dwarfing genes including *dw1*, *dw2*, and *dw3*. This reduced plant heights progressively to 1.5–2.0 m [81,93,154]. These dwarf varieties exhibited significantly improved lodging resistance and enabled a foundation for dense cultivation, with population densities increasing to 18,000–25,000 plants per hectare (plants/ha).

The second transformation in sorghum breeding focused on optimizing population yield (Figure 4), marking a transition from individual plant superiority to population synergy [152]. With the advancement of modern agricultural industrial technology systems, breeding objectives transitioned from focusing solely on large panicles and high grain weight per plant to constructing population types characterized by dwarf and compact size, plant density and stress resistance, and high photosynthetic efficiency. This led to the cultivation of varieties with dwarf size, plant density tolerance, and suitability for mechanized operations, achieving leapfrog improvements in population yield (Figure 4). Molecular breeding technologies further accelerated the synergistic optimization of plant type and population yield [21]. Through marker-assisted selection integrating combinations of dwarfing genes such as *dw1*, *dw3* and *dw2*, *dw3*, plant type was directionally improved, enabling varieties to simultaneously possess traits including dwarf stature with lodging resistance, uniform light penetration within populations, and efficient nutrient utilization.

### 4.2. Major Challenges in Sorghum Plant-Type Breeding

The main cultivated sorghum varieties in China are currently dominated by the medium-dwarf and compact plant type. While the plant type of these varieties generally meets the requirements of high-density planting, they still exhibit limitations in terms of photosynthetic efficiency, lodging resistance, and adaptability to mechanized production.

#### 4.2.1. Plant Type of Main Cultivated Sorghum

The current main cultivated sorghum varieties, predominantly characterized by narrow, upright leaves, are often cultivated using wide–narrow row planting patterns. At appropriate densities, this configuration significantly increases the leaf area index of the population, both improving light transmittance in the middle and lower canopy layers and markedly improving net photosynthetic efficiency. Consequently, average yields have also increased substantially [155]. However, beyond a threshold of 165,000 plants/ha, high-density planting triggers intensified inter-plant competition, manifesting as reduced leaf area per plant, decreased relative chlorophyll content (SPAD values), and reduced photosynthetic capacity in lower leaves. These effects lead to diminishing marginal returns in terms of population photosynthetic efficiency and yield reduction [156].

The lodging resistance of mainstream cultivated sorghum varieties has significantly improved by integrating dwarfing genes and increasing stem diameter. The medium-dwarf plant type led to a decrease in the center of gravity, and the stem diameter coefficient is higher than that of traditional tall-stem varieties [3]. However, under high-density planting, the ventilation between plants will become impeded, stem toughness will decrease, and root systems tend to be relatively shallow, which increase the risk of population lodging under extreme weather [155].

Mechanization adaptability has become a key criterion in sorghum breeding, and the main currently cultivated varieties have undergone targeted optimization of plant type [152]. Plant height is generally controlled below 1.5 m, with loose spikes, fast dehydration, and low threshing loss rate, which meet the requirements of mechanized harvesting [3]. However, some varieties exhibit excessive tillering capacity, which reduces population uniformity and increases the difficulty of mechanical harvesting [157]. Additionally, some brewing sorghum varieties possess relatively short peduncles, which can lead to increased grain damage during mechanical threshing. For sweet sorghum, the juicy nature of the stems poses challenges during mechanical harvesting, as they are prone to clogging equipment. Collectively, these challenges underscore the need for further trait optimization [152].

#### 4.2.2. Gene Pleiotropy and Trait Trade-Offs

The pleiotropic effects of sorghum plant-type genes, together with the negative correlations and trade-offs among various agronomic traits, present a challenge to sorghum breeding [3,158].

This complexity is exemplified by core dwarfing genes, such as *Dw1*, *Dw2*, and *Dw3*, which effectively reduce plant height and enhance lodging resistance while simultaneously exerting associated effects on traits such as panicle type, canopy structure, and root systems [3]. The combined action of *dw1* and *dw3* reduces internode length, while internode number remains constant, resulting in an increased ratio of peduncle length to stem length in dwarf materials, subsequently influencing canopy structure and reducing light transmittance [87]. The *Dw2* locus is additionally linked to *Ma1*, a photoperiod sensitivity locus [159]. Similarly, *Dw3* alleles influence seed number per panicle and seed weight, as well as tiller number and leaf angle [102].

Deterioration in grain quality represents another key consequence of the pleiotropic effects of dwarfing genes [18]. Although *Dw1* and *Dw3* exert relatively weak direct effects on grain size, they influence the efficiency of photosynthate transport, resulting in inadequate grain filling and reduced grain plumpness. Consequently, dwarf varieties carrying these genes generally exhibit lower thousand grain weight than medium–tall-stem varieties. This disparity becomes more pronounced under high-density cultivation conditions, contributing to a significant negative correlation between dwarfing genes and thousand grain weight [160,161].

The trade-off between plant height and grain yield is governed by dwarfing genes. Recessive dwarf alleles (*dw3*) reduce plant height and lodging risk at the expense of kernel weight and yield potential, with limited effects on kernel number [18,95]. Thus, if the goal is to achieve higher grain yield and kernel weight, retaining some plant height is advantageous in sorghum breeding [160,161]. However, an increasing number of studies have shown that dominant tall alleles (*Dw3*) could significantly reduce the plant height of sorghum but had little effect on other important agronomic phenotypes, including single-panicle weight, 1000 grain weight and single-panicle grain number, which provided a theoretical basis for the rational use of the *Dw3* gene in sorghum dwarf breeding.

A recent study identified 22 pleiotropic loci with consistent multi-trait effects, including three major loci on chromosomes 3, 6 (*Dw2*), and 9 (*Dw1*) [158]. Network analysis revealed strong interconnections among loci controlling flowering, plant height, leaf traits, and tiller number [95]. These findings indicate that the trade-off between dwarfing and yield is not absolute but depends on genetic background, allele combinations, and environment. Moreover, QTL analysis showed that while *Dw1* and *Dw3* partially control spikelet-related traits, they are not strongly involved in grain size regulation, challenging the assumption that dwarfing alleles necessarily compromise grain weight [110].

Optimizing planting density to increase population panicle number is a fundamental strategy for enhancing sorghum yield. However, when the planting density exceeds 165,000 plants/ha, ventilation and light penetration among plants deteriorate, stem toughness decreases, root systems have shallower distribution, and the risk of lodging increases significantly [162]. It should be noted that high-density tolerance is a polygenic trait requiring the pyramiding of dwarfing, canopy, root, and stay-green genes, rather than focusing on height reduction alone. Dwarfing alleles (e.g., *dw1* and *dw3*) reduce height and lodging risk, yet significant variation in yield response to increased density exists among semi-dwarf lines, indicating that other factors are involved [1]. Independent crowding resistance traits include erect leaf angle (regulated by Dw3, SbLG1) [102], compact panicle architecture [52], steep root angle (regulated by *qRA1_5*) [60] and stay-green QTLs (*Stg1*–*4*) [46]. GWASs have identified density tolerance loci distinct from height QTLs [101], and shade-avoidance responses occur even in ultra-dwarf lines [122]. In addition, maximizing yield potential under high-density mechanized production often conflicts with maintaining the abiotic stress tolerance (e.g., cold and drought) required for semi-arid and marginal environments, as stress tolerance frequently imposes physiological costs that may compromise yield-related traits [122].

The sink capacity of sorghum panicles is limited. Increasing the grain number per panicle often leads to incomplete grain filling, reduced thousand grain weight, and a higher proportion of non-grain biomass in the panicle [55]. Among the main cultivated varieties in China, specialized brewing types such as Hongyingzi have thousand grain weights of only 22–25 g to maintain grain number per panicle and tannin content, which is 15–20% lower than that of grain-type varieties. In contrast, varieties bred for large grain type often show reduced grain number per panicle, requiring yield compensation through dense planting [51].

Gene pleiotropy is largely determined by gene expression patterns under promoter control. Modifying promoter cis-elements can thus optimize expression and mitigate trait trade-offs, enabling simultaneous improvement in yield, quality, and resistance [163]. Recent genome editing has broken through these trade-off bottlenecks, accelerating the development of multi-trait elite varieties and driving a new wave of crop genetic improvement.

#### 4.2.3. Narrow Genetic Basis of Germplasm Resources

Sorghum germplasm resources are conserved worldwide, spanning more than 100 countries across five continents, with at least 20 gene banks globally, including the National Plant Germplasm System (NPGS) of the United States Department of Agriculture, the International Crops Research Institute for the Semi-Arid Tropics in India, and the Indian National Bureau of Plant Genetic Resources [86], and the National Crop GenBank of China [152].

Despite the preservation of over 240,000 sorghum germplasm accessions in global gene banks, the genetic diversity present in cultivated varieties remains extremely limited, posing a major constraint to advances in plant-type improvement. Modern sorghum breeding relies heavily on a narrow set of core parental lines, such as BTx623, resulting in close genetic relationships among developed varieties and a marked reduction in genetic variation [164]. This issue is particularly evident in China, where the main commercial varieties released over the past decade exhibit a narrow genetic base, lack germplasm tolerance to high-density planting, and have high photosynthetic efficiency [165]. GWASs have revealed that domestication and subsequent improvement have imposed multiple genetic bottlenecks on sorghum, leading to the loss of numerous key alleles governing canopy structure and light interception efficiency. These bottlenecks have also contributed to the expansion of linkage disequilibrium (LD) regions, further restricting opportunities for the recombination of beneficial alleles [166].

The discovery and utilization of superior genes from wild sorghum relatives have progressed slowly [167]. Hybridization incompatibility with cultivated varieties and unstable segregation in progeny populations hinder the effective introgression of desirable traits, such as high photosynthetic efficiency and enhanced stress tolerance, into elite breeding materials [168,169]. For example, cold-tolerant loci and candidate genes have been identified from wild rice, demonstrating how multi-omics can facilitate gene discovery for non-biological stress adaptation [170]. Meanwhile, alleles associated with high photosynthetic rates and strong tolerance to light stress, which are present in wild sorghum accessions, have not yet been successfully transferred into cultivated backgrounds. This is primarily due to low genetic transformation efficiency and persistent issues with linkage drag during introgression [171].

#### 4.2.4. Lack of High-Throughput Phenotyping Technologies

Sorghum plant type is influenced by intricate interactions of genotype, planting density, and environmental factors, making it difficult to simultaneously achieve accuracy and scalability by using traditional evaluation methods. However, large-scale, high-precision phenotyping techniques remain underdeveloped [52,172]. Traditional manual measurements of light interception-related traits using tools such as leaf area meters and photosynthetic instruments are straightforward to implement but are time-consuming, labor-intensive, and destructive. Moreover, they typically yield only single-point, static data, failing to capture the three-dimensional dynamic characteristics of the canopy [173]. Indirect methods, including unmanned aerial vehicle (UAV) remote sensing and near-ground spectroscopy, enable the non-destructive, batch monitoring of canopy reflectance and related parameters. However, these approaches are highly susceptible to environmental fluctuations such as changes in light intensity and weather conditions, and they are difficult to use to accurately quantify internal light distribution or spatial leaf arrangement within the canopy [174,175].

High-throughput phenotyping technologies have overcome several limitations of traditional methods and have been increasingly applied in sorghum for germplasm screening, stress tolerance assessment, plant-type improvement, and yield prediction. However, challenges persist, including insufficient robustness in field conditions, difficulties in analyzing complex traits, a lack of standardized data formats, and high equipment costs [176].

## 5. Future Perspectives: Toward Gene Design Breeding

### 5.1. Gene Design Breeding and Its Workflow

Crop breeding technology has progressed from early domestication and selection, through conventional cross breeding and mutation breeding, to the current era of molecular design breeding, which employs techniques such as marker-assisted selection, molecular design, and genetic modification [177,178].

Marker-assisted selection (MAS) replaces phenotypic assays with DNA markers, improving efficiency and precision [179]. When traditional methods fall short, molecular markers enable marker-assisted breeding (MAB). However, MAS works well for single genes but fails to capture polygenic background effects [180,181]. Genomic selection (GS) addresses polygenic traits, but its sorghum applications are limited by small training populations, high genotyping costs, and insufficient multi-environment validation for ideotype traits [182]. Advanced tools like CRISPR/Cas9 enable precise gene editing, and techniques such as GWAS, MAS, and GS allow for accurate trait selection and outcome prediction [183]. Nevertheless, these tools are still being refined for complex traits shaped by multiple genes and environmental factors [184].

Breeding by design aims to control all agronomically important allelic variation through precise genetic mapping, high-resolution haplotyping, and extensive phenotyping, so it is feasible with current marker technologies and analytics [185]. As a highly integrated, multi-disciplinary system, molecular design breeding enables simulation and optimization before field trials, identifying optimal target genotypes for specific ecological regions and the most efficient crossing/selection strategies to achieve them. This approach shifts the breeding paradigm from “phenotypic breeding by experience” to “genotypic breeding by prediction”, substantially improving predictability, efficiency, and effectiveness [177,180]. It has been widely applied in wheat, corn, rice and sorghum, leading to enhanced crop yield, quality, and resistance [186]. Design breeding consists of three major steps [180,185]: First, identify key genes and their interactions by establishing genetic populations, screening markers, constructing linkage maps, and conducting phenotypic and genetic analyses. Second, define target genotypes for specific breeding objectives and environments through genotype-to-phenotype prediction using gene locations, pathways, expression networks, genetic effects, and gene–gene interactions. Third, devise optimal breeding strategies to achieve the target genotypes, providing a practical blueprint for developing designed cultivars.

Modern breeding is gradually moving toward intelligent breeding, which is grounded in genetics and integrates information technology, gene editing, biotechnological breeding and artificial intelligence approaches. This enables precise design and manipulation at the genetic level, the pyramiding of favorable alleles, and accurate targeted improvement in traits, ultimately allowing for the design and creation of new varieties that are adapted to different environments, possess excellent traits, and meet human needs [187].

### 5.2. Multi-Omics-Driven Gene Discovery

The merger of genomics and phenomics—where genomics refers to the study of an organism’s entire DNA sequence and pheromonics is the full explanation of observable characteristics has given a new face to breeding strategies [188], has enabled the discovery of the genetic variations and excellent gene resources of crop germplasm resources, which is of great significance for molecular design breeding practice.

As a diploid species (2n = 20) with a relatively small genome and broad environmental adaptability, sorghum offers rich genomic resources and extensive phenotypic variation for crop improvement [189]. Early efforts to identify sorghum genotypes primarily relied on traditional molecular markers. While numerous loci associated with agronomic traits were identified, the long breeding cycles inherent in conventional approaches limited the pace of genetic progress in variety development [86]. However, the advent of high-throughput sequencing has substantially advanced the genotypic characterization of sorghum germplasm, greatly enriched genomic data and improved sequence resolution [190,191]. Concurrently, methods such as bulked segregant analysis (BSA) and GWAS have become widespread in dissecting the genetic basis of complex agronomic traits in major crops. These approaches have facilitated the discovery of superior alleles and provided molecular evidence for crop improvement efforts [171,192].

The sequencing of the sorghum reference genome BTx623 provided a critical foundation for subsequent genome annotations [193]. Based on deep sequencing, linkage analysis, and transcriptomic data, the original reference genome annotation was later updated to enhance accuracy and utility [2]. Subsequently, reference genomes for additional sorghum varieties, such as Tx430, have been developed, providing valuable resources for studying domestication and genetic improvement [194]. With the rapid advancement of genomics, technologies such as Hi-C and transcriptomics have been employed to construct the first broadly representative, high-quality sorghum pangenome, offering a robust foundation for domestication research and breeding applications [195]. More recently, complete telomere-to-telomere (T2T) genome assemblies have been achieved for BTx623, Ji2055, and Hongyingzi [196,197,198]. Furthermore, databases such as the transcriptome database (MOROKOSHI), the SNP database (SorGSD), and the multidimensional network analysis platform (SorghumFDB), as well as the applications of proteomics, metabolomics, and phenomics in different fields of sorghum, provide important resources for studying sorghum genetic diversity, the evolutionary relationships between different subspecies and environmental adaptability [199,200]. Sorghum breeding has entered an exciting new era.

### 5.3. High-Throughput Phenotyping Technologies

In recent years, the deep integration of robotics technology, sensor science, and artificial intelligence has enabled the development of a high-throughput phenotyping system that integrates large-scale field collection, precise indoor analysis, and multi-modal data fusion. This system allows for the efficient and non-destructive assessment of key traits such as plant height, canopy structure, panicle morphology, and stem characteristics, thereby providing precise data support for high photosynthetic efficiency breeding and cultivation optimization in dense planting [176,201,202].

Field phenotyping detection is based on UAVs and ground-based robots, enabling large-scale and rapid data acquisition [203,204]. UAVs equipped with RGB cameras and LiDAR, combined with digital terrain models and point cloud reconstruction, achieve plant height measurements with a mean error of only 18 cm compared with manual measurements [205]. Integrating Neural Radiance Fields (NeRFs) with the SegVoteNet model, the accuracy of field spike detection is 0.85, which is suitable for multiple environmental scenarios [206]. Field robots employing Structure from Motion (SfM) technology can complete 3D reconstruction of 72 plants per hour, efficiently extracting parameters such as leaf area and stem diameter and solving the problems of occlusion and variable lighting [207]. Multi-temporal UAV imagery combined with random forest models enables flowering time prediction with an error margin within 2.6 days, supporting the dynamic monitoring of plant type [208,209]. Furthermore, near-infrared spectroscopy (NIRS) coupled with support vector machine regression enables the rapid prediction of stem structural characteristics [40].

Despite these advances, current phenotyping technologies still face challenges such as insufficient environmental adaptability and weak model generalization. These advancements will facilitate the collaborative analysis of genotype–phenotype–environment (G-P-E) interactions and provide efficient technical support for breeding sorghum varieties with high yield, stress tolerance, and mechanical harvest suitability.

### 5.4. Application of Genome Editing Technologies

To achieve improvement in plant-type traits in sorghum, the use of gene editing technologies such as CRISPR to precisely manipulate key genes has become the main driving force for breaking genetic linkage and creating new germplasm [210]. Although molecular breeding efforts in sorghum have lagged behind those for major staple crops, significant progress has been made in genetic transformation and genome editing [211,212]. Regarding genome editing tools, the use of protoplast systems effectively promoted the transient gene expression and editing mediated by CRISPR/Cas9 in sorghum [213] and successfully achieved genome editing in Tx430 using gene gun bombardment [214]. At present, multiple editing systems, such as Agrobacterium-mediated, gene gun, and protoplast transformation, have been established, with editing efficiency of 15–40% and a cycle time of 8–10 months. Targeted gene modification has been achieved through genetic transformation using the CRISPR/Cas9 system [215].

In terms of sorghum improvement and creation, editing using *K2G* [216], *SbBADH2* [217], and *SbMATE* [218] has successfully generated new sorghum varieties with reduced kafirin content, enhanced fragrance, and improved aluminum tolerance, respectively. Looking ahead to the future, genome editing technology offers the potential to directly insert target DNA sequences into the genome, replacing or deleting specific genomic fragments to achieve the desired traits. This capability is expected to propel sorghum breeding into a new stage of design and creation. It will not only greatly broaden the genetic base of sorghum breeding but also fundamentally break unfavorable genetic linkages, enabling the flexible design and assembly of plant-type configurations. Such advances will bring unprecedented precision and predictability to sorghum breeding programs in China.

### 5.5. Strategy for Construction of Sorghum Ideotype

Traditional sorghum hybrid breeding relies on cytoplasmic male sterility (CMS) systems and dwarfing genes to develop high-yielding hybrids. It is time-consuming, suffers from linkage drag and pleiotropic trade-offs, and is inefficient for polygenic traits. In contrast, gene design breeding integrates MAS, GS, CRISPR/Cas9, high-throughput phenomics, and AI-driven models, enabling precise, rapid improvement of target genes, fine-tuning expression, and pyramiding favorable alleles. The future lies in integrating traditional and molecular design breeding, shifting from experience-driven to intelligently designed strategies [219,220].

**Figure 5:**
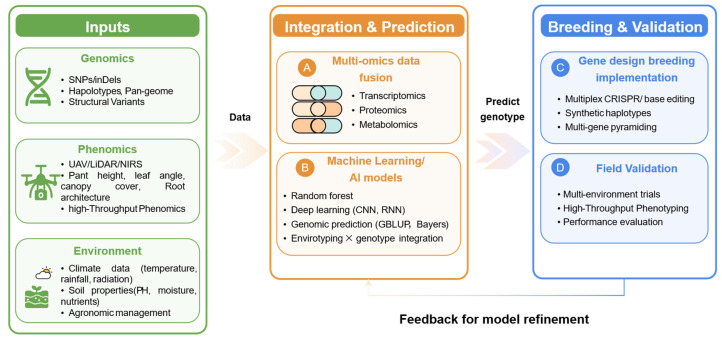
Systems-level integration of multi-omics, phenomics, and artificial intelligence (AI) into a gene design breeding pipeline for sorghum.

Gene design breeding integrates multi-omics, high-throughput phenomics, and artificial intelligence into a unified, iterative workflow (Figure 5). First, large-scale data acquisition involves whole-genome resequencing, UAV-based high-throughput phenotyping and environmental characterization. Second, multi-omics data integration combines genomics, transcriptomics, proteomics, and metabolomics with envirotyping data, leveraging platforms to create a multi-layered molecular and environmental dataset. Third, AI and machine learning models are trained to predict ideotype performance and optimal haplotype combinations. These models capture complex nonlinear interactions across data layers and have been shown to improve prediction accuracy when integrating genomics with transcriptomics. Fourth, breeding implementation translates the predicted optimal haplotypes into assembled genotypes using gene design breeding strategies: multiplex CRISPR/Cas9 for the simultaneous editing of target genes, base editing or prime editing for fine-tuning expression, and synthetic haplotypes to introduce novel allele combinations not present in the germplasm pool. Fifth, field validation and feedback test the assembled lines under multi-environment conditions with high-throughput phenotyping; the resulting phenotypic data are used to retrain and refine the AI prediction models, closing the loop and enabling iterative improvement across breeding cycles.

In future sorghum breeding research, it will be possible to systematically integrate multi-dimensional datasets such as genotypic data (superior haplotypes and allele combinations), high-precision phenotypic data (morphological traits such as plant height, tiller number, and leaf angle), and environmental data (climate and soil characteristics across locations and seasons) based on the key genes and haplotypes already mined. Using machine learning and multivariate statistical analysis, the dynamic relationships between key traits and yield, stress resistance, and environmental adaptability can be quantified, laying the groundwork for predictive G-P-E interaction models. By integrating these data, a computational model for sorghum ideotype can be constructed and refined. Such a model would enable, for example, the input of environmental parameters to generate recommended genotype combinations, thereby guiding both parent selection and progeny screening. Breeding populations typically involve crossing a large number of parents that simultaneously possess multiple desirable traits, with the expectation of generating superior progeny through trait (gene) complementation and transgressive segregation [221]. The progeny materials then undergo strong artificial and natural selection [222]. Therefore, it is necessary to develop novel multi-parent genetic mating designs to create ideal populations that have both breeding value and suitability for genetic studies. During the parental selection stage, the model could simulate the probability of different parental combinations producing offspring with the ideotype, enabling precise recommendation of optimal cross-combinations. During progeny evaluation, the model could predict the ultimate phenotypic performance and yield potential of breeding lines in target environments based on their genotypic profiles, thus facilitating efficient early-stage selection. Finally, this model will promote sorghum breeding from empirical field-based screening to a new era of computer-aided intelligent design, systematically improving breeding efficiency and accuracy.

Gene design breeding has long been used to support ideotype design [223]. In rice, the CERES-Rice model combined with global sensitivity analysis and AI optimization defined an ideotype that maximizes both grain yield and water use efficiency [224]. In wheat, the APSIM Next Generation model, coupled with envirotyping and genetic algorithms, identified ideotypes that improved average yield by approximately 18% and yield stability by up to 16% [225]. In maize, an automated high-throughput phenotyping platform (HTP), integrated with genome-wide association studies and machine learning, identified key genetic loci controlling plant height. This approach enabled efficient, non-destructive assessment of growth dynamics and provided a powerful template for ideotype breeding [226]. Although the application of computational ideotype modeling in sorghum is still emerging, several promising initiatives demonstrate its feasibility. An ecophysiological modeling approach was developed for biomass sorghum using the Ecomeristem plant growth model, which quantifies trait impacts on plant phenotype construction while considering linkages and trade-offs among traits that can modulate their benefits depending on genotype and environment [71]. Furthermore, high-throughput phenotyping tools compatible with genetic analyses and breeding constraints have been purposefully developed to establish a framework that progresses from “industrial ideotypes” to “molecular ideotypes” for multi-purpose sorghum [227].

However, ideotype breeding faces several fundamental limitations. First, yield is a complex polygenic trait, rendering single-trait selection ineffective. Second, optimizing traits across environments requires extensive genotype–environment–management (G × E × M) data. Third, high-throughput phenotyping technologies remain expensive and expertise-dependent, limiting their accessibility. Fourth, transferring desirable alleles from wild relatives or landraces is hindered by linkage drag, hybridization barriers, and negative pleiotropy, leaving many valuable alleles untapped. Fifth, focusing on a narrow set of ideal traits may erode genetic diversity, increasing susceptibility to pests and diseases. Six, in sorghum, the cytoplasmic genetic male sterility (CGMS) system adds complexity, as ideotype alleles may interact unpredictably with mitochondrial sterility factors [228]. Finally, developing a sorghum ideotype suitable for mechanized harvesting and high-density planting is not merely a genetic or breeding challenge, it demands interdisciplinary collaboration and integrated expertise.

Several strategies can make this slow process more efficient. These include rapid generation advance (e.g., speed breeding) to shorten generation time, genomic selection to predict multi-trait performance without extensive field phenotyping, haplotype-based breeding to track favorable allele combinations across generations, and multiplex CRISPR to simultaneously introduce multiple desired alleles into an elite background, bypassing lengthy crossing and backcrossing. Finally, ideotype breeding must integrate abiotic stress adaptation from the outset, using multi-omics platforms to dissect tolerance mechanisms and favorable alleles without disrupting core ideotype architecture. Accordingly, breeding strategies should be tailored to specific production environments and market segments, enabling the development of sorghum ideotypes that are high-yielding under optimal conditions yet resilient to abiotic stresses in marginal environments.

## 6. Conclusions

The realization of an ideotype in sorghum requires the synergistic improvement of the golden triangle comprising efficient light utilization, lodging resistance, and mechanization adaptability. Future breeding efforts will require the deep integration of traditional empirical knowledge with modern molecular design and intelligent phenotyping technologies. By pyramiding multiple genes and enabling intelligent environmental adaptation, we can cultivate new varieties that are high-yielding, stress-tolerant, resource-efficient, and suitable for mechanized production systems. These advances will provide support for global food security and the advancement of sustainable agriculture.

The key to achieving an ideotype in sorghum lies in the coordinated optimization of light utilization, lodging resistance, and mechanization adaptability to create a more efficient and coordinated plant type. Dwarfing represents a common requirement for both lodging resistance and mechanization adaptability, but it can also simultaneously lead to reduced biomass. Therefore, optimizing light utilization requires compensatory strategies, such as developing erect leaf angles and enhancing photosynthetic efficiency. Future advances in the sorghum ideotype will not involve the extreme pursuit of any single trait but rather the systematic integration of multiple traits through polygene pyramiding and the precise balancing of light utilization, lodging resistance and mechanization adaptability. It is essential to transcend the limitations of traditional single-trait improvement by adopting multidisciplinary approaches and modern breeding technologies (e.g., molecular marker-assisted selection and gene editing) to overcome unfavorable genetic correlations among traits. This will advance sorghum breeding from empirical selection to the era of precision design, ultimately enabling the development of next-generation sorghum varieties that are high-yielding, stable in performance, and suitable for mechanical harvesting.

Traditional breeding experience serves as the cornerstone of breeding, embodying the profound understanding accumulated by generations of breeders regarding sorghum agronomic traits, environmental adaptability, and combining ability, ultimately defining the breeding objectives and the very connotations of superior varieties (e.g., tolerance to barren soils and high brewing quality). Modern molecular biotechnology achieves precise identification and manipulation at the genetic level through molecular marker-assisted selection, genomic selection, and gene editing, significantly improving breeding efficiency and accuracy. Meanwhile, plant phenotype, relying on UAV remote sensing, sensor networks, and artificial intelligence, achieves the high-throughput, objective collection of phenotypic data, effectively bridging the gap between genetic information and field performance. Accurate, high-throughput phenotype analysis will accelerate plant genetic improvement and drive the next green revolution in crop breeding. Therefore, integrating traditional breeding experience with modern molecular biotechnology and intelligent phenotyping technologies can effectively break unfavorable genetic linkages between a high-yield and poor-quality ideotype and stress sensitivity. This synergy will significantly accelerate the development of new varieties to meet the diverse demands of sorghum breeding and will be an indispensable pathway for cultivating breakthrough sorghum varieties in the future.

To ensure national food security and agricultural sci-tech self-reliance, a strategic policy framework for sorghum breeding is urgently needed. This requires a top-tier research system led by national laboratories and innovation centers, targeting breakthroughs in germplasm and molecular breeding. Breeding goals should be embedded into national sci-tech plans, prioritizing mechanized, multi-purpose varieties. Project evaluation must shift to market-oriented mechanisms with fault-tolerant and classified systems. A collaborative landscape integrating research institutes and enterprises should be fostered, with enterprises leading market-driven projects, deepening industry–academia partnerships, and enhancing talent incentives. Ultimately, enterprise-led, multi-party innovation consortia will boost the core competitiveness of the sorghum seed industry.

## Figures and Tables

**Table 1:** Comparison of ideotype components of rice, wheat, maize and sorghum.

Trait/Component	Rice	Wheat	Maize	Sorghum
Plant height	0.9–1.1 m	0.7–0.8 m	2.2–2.5 m	Semi-dwarf to dwarf
Tillering	8–10 effective tillers	Few unproductive tillers	Single stalk (no tillering)	Controlled tillering
Leaf angle and canopy	Erect, dark green leaves; compact canopy	Short, broad, thick flag leaves; erect	Upright above ear, horizontal below it	Erect upper leaves, progressively horizontal lower leaves; narrow leaf angle preferred
Panicle morphology	Large panicle with many spikelets, rapid grain filling	Large spike with many grains	Erect ear, moderate placement, compact tassel	Compact panicle, moderate exsertion, loose spike for threshability
Stem strength and lodging resistance	Robust stems, lignin-rich	Strong stems, short thick internodes	Thick stalks	Thick stems, short basal internodes, high lignin content
High-density adaptation	High	Moderate–high	Very high	High with upright leaves and compact panicles
Mechanized harvesting suitability	Good	Good	Excellent	Optimized

## Data Availability

All data supporting this research article are already included in this manuscript.
